# Congenital Mesoblastic Nephroma Mixed Subtype: A Case Report of a Rare Neonatal Tumor

**DOI:** 10.7759/cureus.70704

**Published:** 2024-10-02

**Authors:** Mohammed Alpakra, Sara S Hassanien, Badriah G Alasmari, Abdelhakam A Elmugadam, Ali M Tahir, Mohammed Beaiti, Omar Safar, Mohamed F Bazeed, Mahmoud Hussein

**Affiliations:** 1 Department of Oncology and Hematology, Armed Forces Hospital Southern Region, Khamis Mushait, SAU; 2 Department of Pediatric Hematology/Oncology, Armed Forces Hospital Southern Region, Khamis Mushait, SAU; 3 Department of Pediatrics, Armed Forces Hospital Southern Region, Khamis Mushait, SAU; 4 Department of Urology, Armed Forces Hospital Southern Region, Khamis Mushait, SAU; 5 Department of Radiology, Armed Forces Hospital Southern Region, Khamis Mushait, SAU; 6 Department of Pathology, Armed Forces Hospital Southern Region, Khamis Mushait, SAU

**Keywords:** antenatal detection, congenital mesoblastic nephroma, fetal tumors, magnetic resonance imaging, renal tumor

## Abstract

Congenital renal tumors are rare. In infancy, congenital mesoblastic nephroma is the most commonly reported renal tumor. It is recognized antenatally due to polyhydramnios and presents clinically as a palpable abdominal mass in the neonatal period. Although widely regarded as a benign tumor, radical nephrectomy is routinely adopted as an effective treatment.

We report a case involving antenatal recognition of a large fetal renal mass with polyhydramnios at 35 weeks of gestation. At 37 weeks of gestation, the baby was delivered via emergency cesarean section due to fetal distress. After delivery, a magnetic resonance imaging scan confirmed a solid mass in the left kidney, and a left radical nephrectomy was performed in the second week of life. The subsequent pathological examination confirmed a mixed (cellular and classical) variant of congenital mesoblastic nephroma.

## Introduction

Congenital mesoblastic nephroma (CMN) is the most common kidney tumor in infancy, with a reported incidence of 1:125,000 [1]. The following three variants of CMN have been identified: mixed, cellular, and classic. Cellular CMN is aggressive and capable of metastasis, malignancy, and recurrence. In contrast, classic CMN has a favorable prognosis [2]. An asymptomatic abdominal mass is the most typical presentation; other clinical presentations may include vomiting, hypercalcemia, hematuria, proteinuria, dehydration, and electrolyte abnormalities. Around 67% of cases are characterized by hypertension due to hyperreninemia. Sonography and magnetic resonance imaging (MRI) are the mainstays of diagnosis [3]. Surgical radical nephrectomy is required and carries a favorable prognosis. The pathological type and stage are contributing factors when judging the outcome. Adjuvant chemotherapy is indicated in selected individuals with relapsed, high-stage, late-onset cellular CMN or positive surgical margins [4]. Here, we report a case of an antenatally detected left renal mass during routine fetal scanning at 35 weeks’ gestation.

## Case presentation

**Figure 1:**
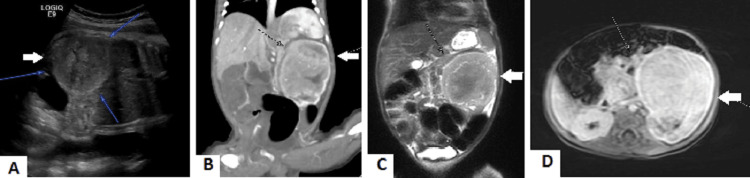
Enlarged left kidney showing a well-defined mass lesion with homogeneous signals and enhancement. (A) Intrauterine fetal ultrasound. (B) Coronal CT of the abdomen. (C) Coronal T2-weighted MRI. (D) Axial MRI.

A left renal mass was detected antenatally during routine fetal scanning at 35 weeks’ gestation in a previously medically free 19-year-old gravida 4 para 3 woman (Figure 1A). The baby boy was delivered by emergency cesarean section at 37 weeks’ gestation due to fetal distress and severe polyhydramnios. His Apgar scores were 8/9/9, with a weight of 2.43 kg, head circumference of 33 cm, and length of 49 cm. His abdomen was distended with a palpable mass in the left upper quadrant measuring 5 × 4 cm. The mass was non-tender, mobile, firm, and had regular margins. There was neither any recorded hypertension nor hematuria. Computed tomography (CT) scan of the abdomen performed three days after birth showed an enlarged left kidney with a solid mass measuring 4.2 × 3.2 × 3.6 cm in the middle zone, with homogeneous enhancement. The kidney significantly distorted the pelvicalyceal system, involving the renal sinus and renal pelvis, and displaced the mesenteric and celiac arteries (Figure 1B). An MRI with contrast revealed a well-defined solid left renal mass with homogeneous signal intensity, without calcification, hemorrhage, cystic components, or necrosis. The mass measured 5 × 5.1 × 5.3 cm, consistent with mesoblastic nephroma as the primary differential diagnosis (Figures 1C, 1D).

An exploratory laparotomy was performed on the 11th day of life. Through a left lower transverse incision, a left radical nephrectomy was carried out. The mass was located inferior-medial to the renal hilum, with elongated renal vessels under control. A smooth, solid, spherical, lobulated mass with multiple feeding vessels on its outer surface was observed. The tumor was completely removed without spillage, and the capsule remained intact. The patient had a smooth postoperative recovery with no intraoperative complications. Feeding was initiated on the second postoperative day.

**Figure 2:**
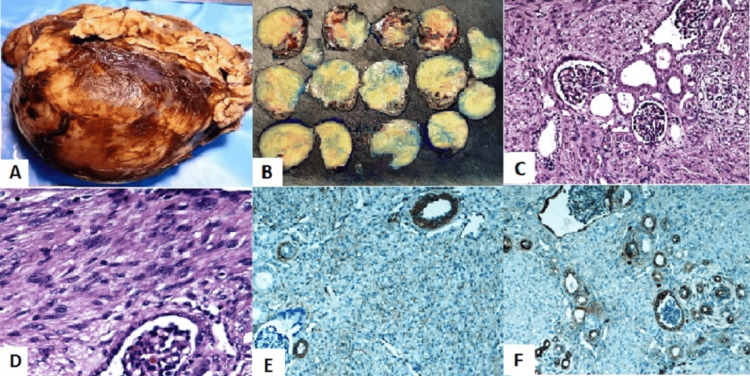
(A, B) Gross features of congenital mesoblastic nephroma, a solid mass, mostly occupying the entire kidney, enwrapped by fatty tissues. Cut sections have a rubbery, myomatous gray appearance with cystic degeneration and hemorrhage. (C, D) Histopathological sections showing residual islands of renal tissue (glomeruli and renal tubules) entrapped by a tumor consisting of intermingling sheets of plump spindle cells, separated by collagen bundles. Neoplastic cells were positive for smooth muscle actin (E) and BCL-2 (F) (magnification C: x200, D: x400, E: x200, F: x200).

The histopathology report described a fully resected unifocal mass measuring 4.5 cm at its largest dimension. The tumor involved the renal sinus fat and invaded focally into, but not through, the renal capsule, with a negative surgical margin (Figures 2A, 2B). The tumor was composed of sharply demarcated spindle cells and primitive round cells. The spindle cell component exhibited low mitotic activity, forming maturing and mature tubules and glomeruli. The round cell component comprised sheets of mitotically active, undifferentiated cells with nuclear polymorphism, irregular nuclear membranes, and difficult-to-discern cytoplasm. Immunohistochemistry revealed that the tumor cells were positive for cyclin D1 and smooth muscle actin (SMA), while WT-1 staining was negative (cytoplasmic). Other negative stains included vimentin, PAX8, CD99, ALK, CD34, and p53. Hence, the diagnosis of CMN, a mixed subtype, was confirmed (Figures 2C-2F).

The management team decided to reserve chemotherapy only in case of recurrence. The baby was scheduled for follow-up in the pediatric oncology department. He remained clinically stable and was thriving well, with routine blood hematology and other chemical results within the acceptable range.

**Figure 3:**
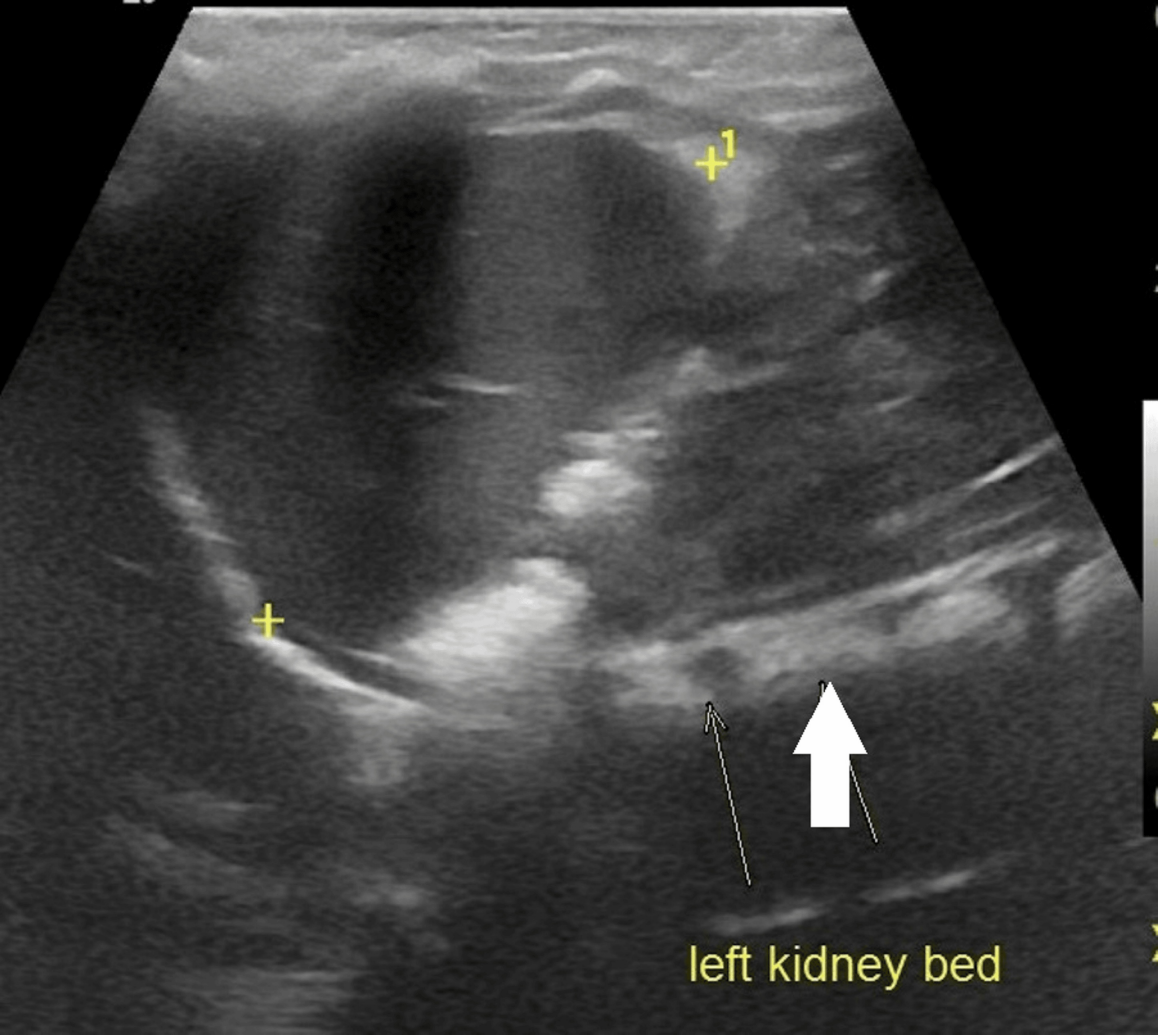
Follow-up post-operative ultrasound showing an empty left kidney bed.

Based on surveillance ultrasonography, the child was seen in the clinic at three months, with no signs of recurrence (Figure 3).

As planned by the pediatric oncologist, long-term follow-up will continue every three months for the first two years after surgery, followed by every six months for the next two years, and then annually until the child is at least five years old.

## Discussion

Neonatal tumors are a rare entity, accounting for 2% of all childhood malignancies. They are a rare phenomenon, reported in one in 12,500-27,500 live births. CMN is the most common renal tumor in infancy. Nevertheless, its incidence is still exceedingly rare [1]. It is a benign mesenchymal renal tumor. Bolande et al. first reported this renal tumor in infancy in 1967, categorizing it histologically as classical, mixed, and cellular subtypes. The classical type (24%) represents a benign tumor, with no cases of recurrence or metastasis documented in the literature. In contrast, the cellular type (66%) inherits the most aggressive nature. The mixed type (10%) bears traits from both categories [5].

CMN can manifest antenatally as polyhydramnios and hydrops fetalis, as well as premature delivery. The clinical picture may include a palpable flank mass, hypertension due to elevated renin, hematuria, and hypercalcemia (paraneoplastic syndrome). Heart failure and pulmonary hypertension may also be encountered [2].

An age greater than three months, positive surgical margins, cellular variety, and tumor rupture during excision are poor prognostic factors [2]. In our case, none of these factors were identified.

The differential diagnosis for a fetal renal tumor includes CMN, Wilms tumor, adrenal neuroblastoma, and clear cell sarcoma. Traditionally, CMN was often confused with Wilms tumors due to multiple similarities. However, CMN is now accepted as a distinct entity, as its benign behavior and lack of malignant epithelial components necessitate pathological differentiation. The histological differential diagnosis of CMN includes metanephric stromal tumor (CD34 positive), stromal type Wilms tumor (WT-1 positive), and inflammatory myofibroblastic tumor (ALK-positive), all of which were negative in our case [4].

The benign course of CMN leads to radical nephrectomy as the primary intervention. Adjuvant chemotherapy is necessary for stage III cellular variants and incomplete tumor resection. Although rare, most recurrences occur within one year of resection. Local recurrence and distant metastases (lungs) have been associated with the cellular subtype [6].

CMN is rarely detected in the antenatal period. There have been approximately 30 reported cases of antenatal diagnosis [2]. Currently, antenatal diagnosis as early as 26 weeks’ gestation is possible. Additionally, contrast-enhanced CT or MRI can help define the tumor’s site of origin, assess its local extent, and rule out distant metastases [7].

## Conclusions

CMN is a rare tumor, usually encountered in newborns and infants under six months of age. Antenatal ultrasound carries a significant importance in detecting congenital renal anomalies. Early diagnosis of a renal mass, especially antenatally, can lead to prompt intervention and a favorable prognosis. Good prognostic factors improve the chance of survival. Surgery is the mainstay of intervention, with chemotherapy reserved for selected patients with risk factors. Vigilant follow-up is essential due to the tendency for local recurrence, primarily in cellular and mixed varieties.
